# Inherited Retinal Diseases Due to *RPE65* Variants: From Genetic Diagnostic Management to Therapy

**DOI:** 10.3390/ijms22137207

**Published:** 2021-07-05

**Authors:** Manar Aoun, Ilaria Passerini, Pietro Chiurazzi, Marianthi Karali, Irene De Rienzo, Giovanna Sartor, Vittoria Murro, Natalia Filimonova, Marco Seri, Sandro Banfi

**Affiliations:** 1 Novartis Farma, Largo Boccioni 1, 21040 Origgio, Italy; manar.aoun@novartis.com; 2 Department of Genetic Diagnosis, Careggi Teaching Hospital, 50134 Florence, Italy; passerinii@aou-careggi.toscana.it; 3 Istituto di Medicina Genomica, Università Cattolica del Sacro Cuore, 00168 Rome, Italy; pietro.chiurazzi@unicatt.it; 4 Fondazione Policlinico Universitario “A. Gemelli” IRCCS, UOC Genetica Medica, 00168 Roma, Italy; 5 Eye Clinic, Multidisciplinary Department of Medical, Surgical and Dental Sciences, Università degli Studi della Campania “Luigi Vanvitelli”, 80131 Naples, Italy; karali@tigem.it; 6 Telethon Institute of Genetics and Medicine (TIGEM), 80078 Pozzuoli, Italy; 7 Department of Ophthalmology, AOU-Careggi, 50234 Florence, Italy; derienzoi@aou-careggi.toscana.it; 8 Medical Genetics Unit, IRCCS Azienda Ospedaliero-Universitaria di Bologna, 40138 Bologna, Italy; giovanna.sartor2@studio.unibo.it; 9 Department of Neuroscience, Psychology, Drug Research and Child Health, University of Florence, Eye Clinic Careggi Teaching Hospital, 50234 Florence, Italy; vittoria.murro@unifi.it; 10 Novartis Pharma AG, Fabrikstrasse 6, CH-4002 Basel, Switzerland; natalia.filimonova@novartis.com; 11 Department of Surgical and Medical Sciences, University of Bologna, 40138 Bologna, Italy; 12 Medical Genetics, Department of Precision Medicine, University of Campania “Luigi Vanvitelli”, 80138 Naples, Italy

**Keywords:** inherited retinal diseases, *RPE65*, next-generation sequencing, variants of uncertain significance, genetic testing, genetic counseling

## Abstract

Inherited retinal diseases (IRDs) are a heterogeneous group of conditions that include retinitis pigmentosa (RP) and Leber congenital amaurosis (LCA) and early-onset severe retinal dystrophy (EO[S]RD), which differ in severity and age of onset. IRDs are caused by mutations in >250 genes. Variants in the *RPE65* gene account for 0.6–6% of RP and 3–16% of LCA/EORD cases. Voretigene neparvovec is a gene therapy approved for the treatment of patients with an autosomal recessive retinal dystrophy due to confirmed biallelic *RPE65* variants (*RPE65*-IRDs). Therefore, the accurate molecular diagnosis of *RPE65*-IRDs is crucial to identify ‘actionable’ genotypes—i.e., genotypes that may benefit from the treatment—and is an integral part of patient management. To date, hundreds of *RPE65* variants have been identified, some of which are classified as pathogenic or likely pathogenic, while the significance of others is yet to be established. In this review, we provide an overview of the genetic diagnostic workup needed to select patients that could be eligible for voretigene neparvovec treatment. Careful clinical characterization of patients by multidisciplinary teams of experts, combined with the availability of next-generation sequencing approaches, can accelerate patients’ access to available therapeutic options.

## 1. Introduction

Inherited retinal diseases (IRDs) are a group of conditions that lead to a progressive loss of vision and have a combined prevalence between 1:3000 and 1:4000 [1,2]. IRDs are characterized by heterogeneity of genetic causes and phenotypic presentations [1,2]. Retinitis pigmentosa (RP) is one of the most common IRDs [3]. In RP, the loss of photoreceptors, primarily in the peripheral retina, results in the appearance of pigment deposits called bone spicules [4,5]. Depending on age at disease onset, severity, rate of progression and presenting phenotype, the most common IRDs associated with severe visual impairment in childhood are Leber congenital amaurosis (LCA) and early-onset severe retinal dystrophy (EO[S]RD), characterized by severe visual loss from birth or early infancy, wandering nystagmus, amaurotic pupils, and markedly reduced or non-recordable full-field electroretinograms [6,7].

Mutations in more than 250 different genes have been so far implicated as the cause of IRDs [1,2,8]. Among the genes responsible for IRDs, *RPE65* gene variants can cause RP (RP20, OMIM # 613794) and LCA/EORD (LCA2, OMIM # 204100) [7,9,10].

The natural history of *RPE65*-IRDs is variable, with a typical onset between birth and five years of age [11,12]. Typically, patients with *RPE65*-related IRD display a significantly altered visual behavior (light staring with profound nyctalopia, nystagmus) [7], severely reduced or nondetectable fundus autofluorescence to 488 nanometers with relatively normal fundus appearance [13], absent electroretinogram or a residual 30Hz flicker response [14], and a low hypermetropic or myopic refractive error [10]. While some patients have normal or near-normal visual acuity (VA) at young ages, it becomes distinctly impaired in the first decade of life. VA then starts to deteriorate around 15–20 years of age, and declines more quickly after the age of 20. In many patients, the VA worsens to the levels of legal blindness (VA = 20/200) around the age of 16 years [12]. By the fourth decade, all patients in this report are legally blind, and many have no light perception (complete loss of vision). Milder disease phenotypes have been described in individuals with hypomorphic *RPE65* alleles [15,16,17,18]. In particular, unusual phenotypic manifestations of biallelic *RPE65* mutations were detected in four families with fundus albipunctatus-like changes and in one family with high hyperopia [19]. 

In the vast majority of cases, *RPE65*-IRDs are autosomal recessive diseases. However, autosomal dominant inheritance pattern has been proposed by some authors in a small proportion of cases. [20,21,22].

In 2017, voretigene neparvovec (Luxturna^®^, Spark Therapeutics, Philadelphia, PA, USA) gene therapy was approved by the US Food and Drug Administration for the treatment of patients with confirmed biallelic *RPE65* mutation-associated retinal dystrophy and viable retinal cells [23]. A year later, voretigene neparvovec was approved by the European Medicines Agency for a similar indication: the treatment of adult and pediatric patients with vision loss due to inherited retinal dystrophy caused by confirmed biallelic *RPE65* variants and who have sufficient viable retinal cells [24].

This article aims to review the evidence for the genetic basis of *RPE65*-IRDs with a particular focus on the most appropriate approaches to molecular testing of patients that represent possible candidates for the *RPE65*-gene supplementation therapy.

## 2. Epidemiology of *RPE65*-IRDs

It is estimated that there are over 2.5 million individuals affected by IRDs worldwide [25], with RP being the most common form. The prevalence of RP is approximately 1:4000 [3,26,27]. In particular, between 21% and 54% of IRD patients have RP [28,29]. On the other hand, the prevalence of LCA/EORD ranges from 1:30,000 to 1:80,000 [10,30]. LCA/EORD accounts for approximately 5% of all IRDs, with reported numbers ranging from 2.59% to 22% [28,29,30].

It is difficult to accurately estimate *RPE65* mutation prevalence rates, as previous studies included different patient populations, initial diagnoses, or assessment methods, and lack a common denominator (Table 1).

*RPE65* variants are responsible for 0.8–1.5% of IRD cases [34,39,40]. In the US, the frequency of people with *RPE65* mutations was estimated to be 1:576,667, for a total of 563 people with such variants at any given time, or seven new cases per year [32]. An analysis of genotype data from six major world populations predicted that there are 15,620 individuals with biallelic *RPE65* mutations, and that more than 60% of these will be from the African population [33].

*RPE65* mutations account for approximately 0.6–6% of RP cases and for 3–16% of LCA/EORD cases depending on the analyzed cohort [9,10,19,29,31,36,37,38,41,42]. The largest European study on the epidemiology of IRDs, which analyzed the genetic landscape in a cohort of 6089 (4403 families) IRD patients in Spain, identified *RPE65* mutations in 3% of families with autosomal recessive RP [38]. One Italian study reported *RPE65* variants in 8.4% of patients with LCA/EORD [31], which was higher than the published prevalence of *RPE65* mutations in patients with LCA/EORD from countries in Northwestern Europe (1.7–6.1%) [6,43]. This could be because, in the Italian study, patients were preselected for clinical characteristics of *RPE65*-associated disease, namely the diagnosis of LCA/EORD. Indeed, a later Italian study identified *RPE65* mutations in 3.4% of IRD patients [41], whilst the latest publication reported a 2% prevalence of *RPE65* mutations in patients with autosomal recessive RP [37]. According to the authors’ experience with a vast collection of patients from across the country, biallelic *RPE65* mutations account for approximately 1–2% of all IRD patients referred for genetic testing in Italy, with 4–5 new cases identified every year (unpublished data). Recently, the European Vision Institute Clinical Research Network (EVICR.net) performed a multinational survey to understand, amongst other issues, the distribution of *RPE65*-IRDs in Europe. In this survey, three-quarters of responding Italian centers reported following 1–5 patients with *RPE65*-IRD and one-quarter reported following a total of 6–10 patients [44].

## 3. Molecular Biology of *RPE65*

In humans, the *RPE65* gene is located on chromosome 1 (1p31), spanning over 20 kb [26]. *RPE65* includes 14 exons and encodes the retinal pigment epithelium-specific 65 kDa protein (RPE65) [45,46], denominated retinoid isomerohydrolase RPE65 by the current nomenclature. RPE65 consists of 533 amino acids [45]. It is a highly conserved protein expressed at high levels exclusively in the retinal pigment epithelium (RPE) [45]. There are two forms of RPE65, a membrane-bound form (mRPE65), a major form with palmytoylation, and a soluble form (sRPE65) [45,47]. RPE65 is involved in the visual cycle, a multi-step process through which light entering the eye is converted into electrical signals transmitted to the brain. When light reaches photosensitive pigments in the retina, it converts 11-*cis*-retinal to all-*trans*-retinal. RPE65 is an isomerase that re-converts all-*trans*-retinyl ester to 11-*cis*-retinol, ready for a new photoisomerisation event [45]. The absence of RPE65 causes a decrease in 11-*cis*-retinol levels and the accumulation of retinyl esters in the RPE [45]. 

## 4. Sequence Variants in the *RPE65* Gene: An Overview

Only patients with biallelic mutations and viable photoreceptor cells are eligible for gene therapy. Gene therapy procedure for *RPE65*-IRDs involves bilateral vitrectomy followed by subretinal injection [48]. Because of the risks associated with such a procedure, it is important to establish the pathogenicity of the underlying mutations conclusively and whether mutations are biallelic. Genetic diagnosis is critical to ensure that patients fulfil the eligibility criteria for treatment [49]. A key scope of the genetic diagnostic workup in IRDs is to identify which genotypes should be classified as pathogenic and, thus, ‘actionable’ (i.e., likely to respond to the approved gene supplementation therapy), a concept parallel to that of ‘actionable’ mutations in cancer for which targeted therapies exist [50]. The presence of many complex and uncertain variants underscores the importance of undertaking exhaustive genetic screening.

### 4.1. Variants

*RPE65* variants were first linked to LCA/EORD in 1997 [51,52]. The heterogeneity of variants encountered was immediately apparent with Gu et al. reporting five different variations (a missense mutation [p.Pro363Thr], two point mutations affecting splicing and two small re-arrangements [51]), and Marlhens and colleagues describing two mutations (a single nucleotide deletion [c.1056delA] and missense mutation [p.Arg234*] [52]). Since then, a myriad of variants has been discovered, the pathogenicity of which have not always been established.

Parallel to variant discovery, variant classification within the spectrum of clinical significance continues to evolve. In 2015, the American College of Medical Genetics and Genomics (ACMG) and the Association for Molecular Pathology issued guidelines that introduced standard terminology to group variants into five categories (“benign”, “likely benign”, “uncertain significance”, likely pathogenic”, “pathogenic”) and described the process of category assignment based on the available evidence (e.g., population, computational, functional, and segregation data) [53].

As of January 2021, more than 300 variations in the *RPE65* gene are listed in the ClinVar database [54], of which approximately 65 are considered pathogenic, 40 likely pathogenic, and 100 of uncertain significance. Most variations (>260) are single-nucleotide changes [54]. Of note, the sample represented in ClinVar may be biased in favor of pathogenic variations since benign and likely benign variants are rarely reported in public databases. At the same time, 206 *RPE65* variations are listed in the Leiden Open Variation (LOV) Database, 102 of which classified as pathogenic or likely pathogenic and 29 as being of uncertain significance [55]. The Genome Aggregation Database (gnomAD) entry for *RPE65* contains 120 synonymous single-nucleotide variants (SNVs), 284 missense SNVs and 24 SNVs marked as “putative loss-of-function” [56].

The variant prevalence and characteristics of *RPE65* were evaluated in 2240 IRD patients in a laboratory certified by Clinical Laboratory Improvement Amendments in the USA [39]. Eighteen patients (0.8%) had *RPE65*-associated disease, of which 12 (67%) had at least one loss-of-function variant. Of the 35 variants identified in the study, two (5.7%) copy number variants (CNV; one single exon deletion and one deletion of the entire *RPE65* gene) were found [39].

While older studies reported no correlations between specific *RPE65* genotypes and phenotypes [57] or clinical course [58], a very recent study suggests that there is a relationship between mutation type and the age of disease onset [59]. Patients carrying two missense alleles showed a later disease onset (≥1 year of age) than those with one or two truncating variants (<1 year of age; Log Rank test *p* < 0.05) [59].

### 4.2. Variants of Uncertain Significance Assessment

Variants of uncertain significance (VUS) pose a serious challenge in determining the eligibility to gene therapy in *RPE65*-IRDs [48]. Nevertheless, Mahajan et al. reported no correlation between variant subtype or ACMG classification and treatment response. Seven out of 29 patients from their series of patients with a confirmed genetic diagnosis of biallelic *RPE65* gene variants had at least one VUS, and all of them, including three patients with two VUSs, responded to gene supplementation therapy [60]. 

Different approaches can be deployed to assess the pathogenicity of a VUS in the *RPE65* gene. These include extended segregation studies, evaluation of the phenotype, in silico tools to predict protein conservation and functionality, and in vitro functional studies. Moreover, extended targeted or exome sequencing can be used to exclude the implication of other IRD genes in disease etiology. 

Segregation studies of all available relatives can be helpful in determining and reclassifying the pathogenicity of VUSs. Extended segregation analysis showed that the p.Phe83Leu and p.Gly187Glu VUS variants were found in families with IRDs: the former in five LCA/EORD patients from four unrelated families and the latter in seven patients from three unrelated families. Moreover, the two variants were more frequent in patients with LCA/EORD than in patients with other IRDs. To further confirm their pathogenicity, the authors consulted eight gene variant databases and deployed 16 computational algorithms to analyze the putative variant impact on protein functionality, 15 of which identified the two variants as damaging. The overall classification of p.Phe83Leu and p.Gly187Glu was thus changed from VUS to likely pathogenic [61].

In the ACMG guidelines, a patient’s phenotype highly specific for the disease and a fitting family history in diseases with monogenic etiology was considered a supporting criterion for classifying pathogenic variants (the PP4 criterion) [53]. The subsequent guidelines published by the UK’s Association for Clinical Genomic Science state that in some cases it may be appropriate to use PP4 at a moderate or strong level after excluding that other genes are implicated [62]. In the case of *RPE65*-IRDs, the identification of a compound heterozygous genotype with one VUS in a patient with a typical LCA/EORD phenotype (as detailed in the Introduction) in the absence of any alternative genetic cause, would be supportive of a VUS reclassification to the ‘likely pathogenic’ category, especially, in the presence of appropriate allele segregation and in silico prediction.

In silico pathogenicity prediction tools are generally integrated into the bioinformatic pipelines used for the analysis of next-generation sequencing (NGS)-based genetic testing. While useful, bioinformatic predictive methods (e.g., Polymorphism Phenotyping v2 (PolyPhen2), Sorting Intolerant From Tolerant (SIFT), or Deleterious Annotation of genetic variants using Neural Networks (DANN), to name just a few tools), and modeling are not as significant in clinical practice (for a detailed list of tools see Richards et al., 2015 [53]). Philp and colleagues have developed and validated an algorithm termed “estimate of pathogenic probability” (EPP) that predicts the pathogenicity of VUS based on its prevalence, segregation and predicted effects on protein structure [63]. In addition, Iancu and colleagues have recently developed a strategy for the reclassification of VUS that considers five pathogenicity predictors, resulting in an algorithm able to correctly reclassify approximately 70% of VUS as pathogenic in validation datasets [64]. The ACMG algorithm has been updated to incorporate new evidence. Clearly, the classification of VUS is a dynamic process that will continue to change as new data are acquired, stressing the importance of knowledge-sharing in the field.

In vitro studies are only performed in a research setting because they are labor-intensive and costly. They assess the consequences of mutations on protein abundance, localization and function, as well as the impact of splice site variants. A minimal in vitro visual cycle system in 293-F cells to assess the isomerization activity of *RPE65* [65] combined with in vitro mutagenesis was recently used to ascertain the pathogenicity of *RPE65* VUSs prior to gene therapy. The results showed that the p.Gly104Val and p.Pro467Ser protein VUSs were catalytically inactive; indeed, affected patients underwent treatment and responded [48]. This example shows that enzymatic activity assessment in conjunction with in vitro mutagenesis may be useful to determine the pathogenicity of VUSs.

### 4.3. Challenging RPE65-IRD Cases

Although *RPE65*-IRDs are monogenic conditions, some authors have cautiously suggested the possibility of a “double-hit” IRD etiology or a phenotype-modifying effect of co-existing mutations [58]. More work is needed to confirm or reject such a hypothesis and to understand its impact on gene therapy eligibility.

To confirm the phase of putative compound heterozygous variants, both for accurate genetic counseling and, consequently, to confirm eligibility for gene therapy, a segregation analysis of proband’s parents is required. Some patients appear to have a homozygous *RPE65* mutation when first tested. If the patient is homozygous for a known variant and the phenotype points to an *RPE65* etiology, the need for segregation may be, in principle, less compelling. Nevertheless, segregation analysis of ‘homozygous’ variants is always recommended for a comprehensive definition of the genotype. Apparent homozygosity can be underlain by three scenarios: copy number variations leading to loss of heterozygosity [39,66], uniparental isodisomy [67], or true homozygosity due to the existence of genetic isolates, i.e., situations in which both parents carry the same mutation (more probable in isolated locations with a degree of inbreeding or in consanguineous families such as that described by [51]). Another possibility is that of encountering patients who carry two variants in *cis* rather than in *trans*, i.e., on the same allele, with the other allele being wild type. Such patients are not eligible for gene therapy for autosomal recessive diseases. Segregation studies and haplotype resolutions using long reads are an approach of choice to assign genetic variants to the homologous paternal and maternal chromosomes [68].

Clearly, the collaborative efforts of international networks of experts in IRD genetics and the regular updating of public variant databases are highly recommended and will significantly contribute to VUS classification and unsolved case interpretation.

## 5. Testing Strategy

Genetic testing is the only way to make an accurate diagnosis of *RPE65*-IRD and qualify the patient for gene therapy. The complexity of genetic testing can be addressed by forming multidisciplinary teams of experts knowledgeable about IRD genetics and variant interpretation to provide the best care for the patient. In Italy, and specifically in IRD genetics expert laboratories, these teams commonly comprise molecular biologists, laboratory technicians, and medical geneticists, and also include ophthalmologists, bioinformaticians, molecular geneticists and genetic counsellors. In fact, to confirm the pathogenic potential of novel genes or variants, the diagnostic expert knowledge and basic science know-how must be combined to resolve the genotype, even in the most complex cases. A genetic diagnosis is necessary to fulfil the eligibility criteria as described in the recent consensus paper by the Italian IRD Working Group [49]. The stepwise procedure we describe here is a way of increasing genetic diagnostic sensitivity to assign a genetic diagnosis to most patients as shown by Stone at al., 2017 [32]. Comprehensive genetic testing in rare eye disorders (RED) is promoted by the ERN-EYE network, who emphasize the clinical need and relevance of genetic testing in RED [69]. 

The choice of the testing strategy and the interpretation of variants are heavily influenced by the information that the requesting clinician provides to the laboratory. The clinician must communicate an accurate clinical diagnosis and pedigree information. Ideally, the request for a genetic test should also be accompanied by the general anamnesis, ocular anamnesis, including a description of retinal and extra-retinal clinical manifestations (indicative of a syndrome), the family pedigree showing affected family members and pattern of inheritance, mention of consanguinity. This information will enable the correct phenotype–genotype correlations to be made and will point towards syndromic or non-syndromic disease. 

Advances in technology allow for the detection of the underlying genetic cause in up to 76% of patients with IRDs [32]. Genetic testing for *RPE65*-IRDs can be performed by Sanger sequencing, especially when NGS methodologies are not available. However, in order to provide comprehensive testing, we believe it is necessary to include other IRD genes [32]. This is particularly valid in patients with VUS or when assessing eligibility to gene therapy [49]. In these cases, the extended panel sequencing is highly recommended. Briefly, targeted panel-based methods are fast and cheap, but, in the case of negative findings, give no alternative genetic diagnosis. Whole-exome sequencing (WES) allows for the identification of variants in all coding sequences and whole-genome sequencing (WGS) anywhere in the genome, albeit they cost more and require longer and more difficult analysis than sequencing of a panel of genes.

In the clinical setting, a diagnostic laboratory must be certified, fitted with the latest equipment and experienced in state-of-art technologies (such as NGS, WES, multiplex ligation probe amplification (MLPA), or Sanger sequencing) to guarantee the quality of diagnostic reports. The choice of test strategy also depends on the balance between cost, turnaround time, coverage and data storage capacities. Most commonly, the frontline technologies for accurate genetic diagnosis include an ocular NGS panel or clinical exome sequencing (CES; also used to diagnose other conditions) followed by targeted in silico analysis restricted to genes implicated in IRDs. The analysis of the remaining genes is performed only when the “virtual ocular panel” yields no genetic diagnosis and has the potential of discovering new genes responsible for the IRD phenotype. Such an approach could even reduce the time needed for genetic testing, as there is no need to perform a new sequencing but rather to re-analyze the already available data.

The standard turnaround time for targeted testing is around 3–4 months. In patients in whom IRD-specific panels were used to no avail or in those with atypical phenotypes, the strategy foresees the use of CES or WES [70], or CES followed by WES [71]. Instead, WGS should be used in patients who have a phenotype that corresponds to a specific condition, but in whom no mutations were detected in the genes previously identified as responsible for that condition or those unresolved by WES [70,72]. For instance, in patients with a single (‘monoallelic’) pathogenic variant in *RPE65* and a convincing clinical picture (RP or LCA/EORD), the sequencing of the entire gene is warranted to look for CNVs or complex structural variants, SNVs in noncoding regions (UTRs, promoter and potential regulatory elements), and deep-intronic (DI) variants. The number of cases with monoallelic *RPE65* pathogenic variants is rather low, and this approach is mainly used in a research context. On the contrary, DI variants constitute 2% of unique and 4% of all *ABCA4* gene variants implicated in the pathogenesis of another autosomal recessive IRD, Stargardt disease. *ABCA4* gene mutations being more common than those of *RPE65*, it is not surprising that the contribution of DI variants emerged more readily in the pathogenesis of Stargardt disease [73]. As the costs and turnaround times of sequencing technologies decrease, and if the capacity to interpret the functional consequences of noncoding variants increases, the use of WGS in IRD diagnosis will become the method of choice in the clinical setting. Results confirmation is still performed by Sanger sequencing. The EVICR.net survey shows that most responding centers in Europe use clinical-grade tests for diagnosing IRDs; few combine clinical- and research-grade approaches. The most commonly deployed methodologies were IRD-specific gene panels (67%), WES (49%) and diagnosis-directed Sanger sequencing (41%). At present, 41–80% of the IRD patients are genetically solved in 69% of responding EVICR.net centers, and only 5% of the centers obtain a genetic diagnosis in 81–100% of their IRD patients [74].

One drawback of NGS panels is that it is not usually possible to determine CNVs, for which MLPA analysis should be used, particularly if extended segregation analysis is not possible (e.g., the proband is an adopted single child). In a study of 677 individuals with retinal dystrophy, indications of a deletion or duplication seen in NGS data were confirmed with MLPA, qPCR, or chromosome microarray [75].

**Figure 1:**
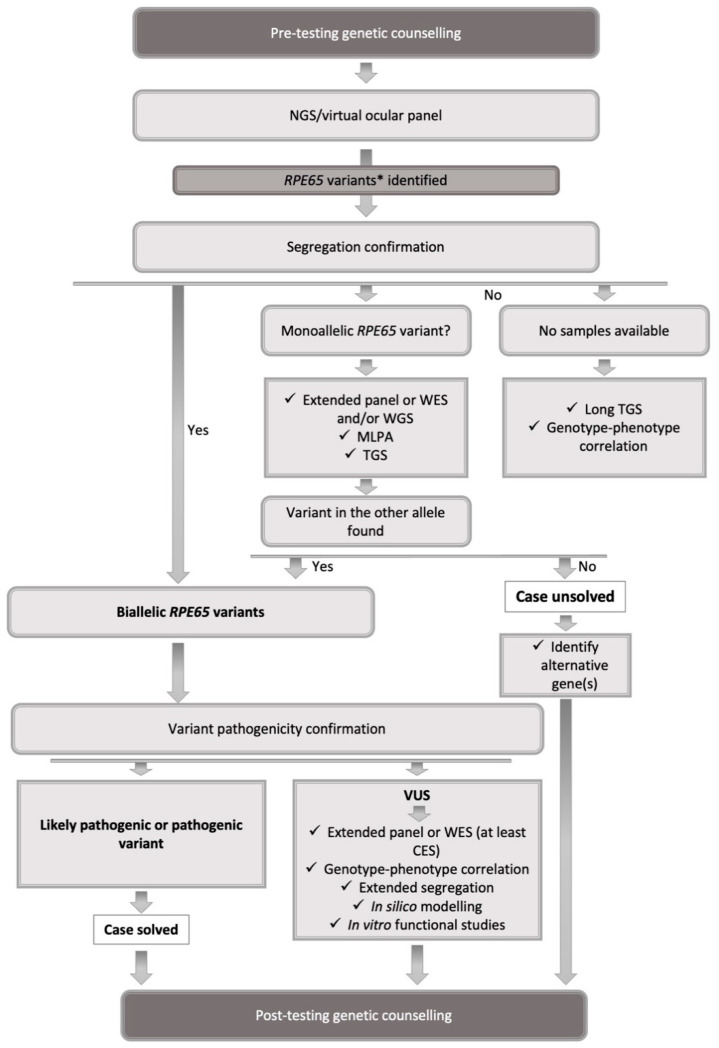
The summary of a proposed genetic diagnostic workup. * If benign or likely benign variants found, the search for a mutation(s) responsible for the phenotype should be extended to other genes. CES, clinical exome sequencing; MLPA, multiplex ligation probe amplification; NGS, next-generation sequencing; VUS, variant of uncertain significance; WES, whole-exome sequencing; WGS, whole-genome sequencing.

Segregation analysis is a fundamental element of the genetic testing strategy as discussed in the section on Sequence variants. Therefore, it is important to acquire samples from parents at the time of the proband’s genetic testing. Although up-front analysis of three samples (trio analysis) rather than one increases the costs of testing, it may well become a standard procedure as costs come down. Figure 1 summarizes a proposed genetic diagnostic workup in *RPE65*-IRDs.

With the advent of third-generation sequencing (TGS) based on the generation of long reads, routine obtainment of reads of >10 kb became possible. Two technologies play prominent roles on the market: single-molecule real-time sequencing known as PacBio (Pacific Biosciences) and nanopore sequencing (Oxford Nanopore Sequencing) [76]. While PacBio is based on recording fluorescence events corresponding to single nucleotide incorporation [77], nanopore sequencing measures ionic current changes when native single-stranded nucleic acid molecule passes through a nanopore [78]. Long reads require apposite analysis tools, a database of which has recently been created [76]. TGS is particularly suitable for genome assembly studies and for the identification of structural variants (i.e., mutations that affect more than 50 base pairs), as reviewed in Xia and Zhou, 2020 [79]). In the context of genetic testing in IRDs, TGS will contribute to the unravelling of orphan cases (lack of segregation data) or in the above-mentioned cases of apparent homozygosity to understanding the *cis*/*trans* allelic phase.

In the future, artificial intelligence (AI) may speed up the genetic testing process, improve the prediction of pathogenicity and the rate of resolved genetic diagnoses. AI approaches in clinical genomics target tasks that are time-consuming and error-prone using traditional methods. Accurate variant calling and classification, genome annotation, functional modelling, and phenotype-to-genotype correspondence—or perhaps future genotype-to-phenotype prediction from genomic data alone—will increasingly be performed using AI [80]. So far, deep neural networks have been used to successfully predict the causative IRD gene in macular dystrophy caused by *ABCA4* and *RP1L1* gene aberration compared with RP caused by *EYS* gene aberration and normal subjects from spectral domain-optical coherence tomography data [34,81]. An increase in diagnostic yield will ultimately improve the rates of patients’ inclusion in clinical trials that lead to the approval of novel treatment options.

## 6. Role of Genetic Counseling

Genetic counseling is defined as “the process of helping people understand and adapt to the medical, psychological and familial implications of genetic contributions to disease” [82]. Importantly, in addition to educating patients about the genetic aspects of their condition, genetic counseling includes elements of psychotherapy [83]. Genetic counseling appears to be effective in educating patients about their condition, providing a greater sense of control and improving how patients assess and manage risk [84].

For patients with IRDs, genetic counseling benefits from both experiences on eye disorders and from an in-depth knowledge of genetic aspects. It is therefore highly recommended that genetic counsellors have experience in ocular genetics or work in multidisciplinary teams with expertise in the field; in particular, counseling sessions conducted jointly by an ophthalmologist and a medical geneticist should be advocated [74].

During genetic counseling scheduled prior to the test, patients have to grant informed consent for testing upon acquiring in-depth knowledge on the range of possible outcomes, the meaning and limitations of the test (such as the fact that in tests evaluating multiple genes, not all variants have a unique interpretation and that testing can produce an uncertain or unresolved outcome), logistical difficulties and waiting times. Also, implications for the family must be explained, including inheritance pattern and the possibility of prenatal diagnosis. Last but not least, counsellors have to address the issue of incidental findings, especially when whole-exome/genome testing is planned. An in silico (virtual) NGS panel approach excludes the possibility of incidental findings as only IRD genes are tested. However, if WES or WGS sequencing is used and incidental findings are encountered, the role of a genetic counsellor is crucial to address their consequences with patients and their families. A number of factors may make patients resistant to genetic testing, including ethical concerns, paternity issues, reluctance to involve family members and lack of information. Some patients fear being told that there is no cure (not so in *RPE65*-IRDs); there may be irrational guilt in parents who feel responsible for passing on the disease, and fear of a genetic disease and denial also play a role.

Communication with patients and their relatives is a delicate, albeit crucial, issue. Healthcare professionals need to take adequate time for genetic counseling (an average session lasts approximately 45 min) and patients have to feel free to talk about their expectations, voice their doubts and be able to contact the professional in case of necessity. Sometimes several consultations are needed to enable patients to understand their conditions, feel empowered and make informed decisions. As many *RPE65*-IRD patients are young children, talking to their parents/careers may prove harrowing for all participants of a counseling session.

When test results are available, genetic counseling should concentrate on interpreting the results obtained, such as variants relevant and not relevant to the phenotype, identification of VUSs or unexpected diagnosis; in case of an uncertain result, the need for further analysis and involving relatives may be described. Post-test genetic counseling also discusses disease management (multidisciplinary care for syndromic patients) and therapeutic options, clinical trial inclusion, or the possibility of testing other family members. If additional genetic workup is needed, the necessity of a further wait for a genetic diagnosis must be raised. As segregation analysis is often a key element in molecular diagnosis, patients with advanced IRDs and no family members for segregation analysis constitute a particular challenge for both genetic testing and counseling.

## 7. Conclusions

Gene therapy is now available for the treatment of *RPE65*-IRDs. Therapies specific for other genotypes are being developed, and clinical trials of their safety and efficacy are underway [85]. Only patients with a sufficient number of viable retinal cells can truly benefit from the therapy. Therefore, a timely and effective genetic testing strategy must be implemented to identify patients eligible for gene therapy by adopting an exhaustive stepwise procedure so that no patient is left without a genetic diagnosis. Furthermore, to ensure the best outcomes for patients, genetic counseling programs should be implemented. As phenotypes and genotypes frequently do not correlate, multidisciplinary teams involving clinicians, geneticists, counsellors, diagnostic laboratories, and basic researchers should form to study the relationships between them and to unravel the molecular/pathogenic mechanisms of each variant to classify VUSs correctly. Frequent interaction, continuous feedback, and collaboration will optimize patient management and maximize the benefit for patients from available treatments.

## Figures and Tables

**Table 1:** Epidemiology of Leber congenital amaurosis, retinitis pigmentosa, and *RPE65*-inherited retinal diseases.

Study	Participants *	Findings *
Simonelli et al. 2007 [31]	Italian patients with LCA/EORD N = 95	*RPE65* mutations in sample: *n* = 8 (8.4%)
Stone et al. 2017 [32]	Consecutive patients with IRD seen by a single physician N = 1000	*RPE65* mutations in the sample: *n* = 3 Estimated frequency in the US general population: 1:576667
Avela et al. 2019 [28]	Finnish children with IRD N = 68	RP: *n* = 14 (21%) LCA/EORD: *n* = 15 (22%)
Chung et al. 2019 [12]	Patients with IRD due to biallelic mutations in *RPE65* N = 70	LCA/EORD: *n*=39 (50.0%) RP: *n* = 6 (7.7%)
Hanany et al. 2020 [33]	Genotype data on six main world populations	Autosomal recessive IRD: 1:1380 RP: 23% LCA/EORD: 7% Biallelic *RPE65*: *n* = 15,620 individuals or 0.3% of all patients with IRD
Holtan et al. 2020 [29]	Norwegian patients with IRDN = 866 N = 685 (living in the south-east of Norway)	RP: *n* = 468 (54.0%) LCA/EORD: *n* = 45 (5.2%) *RPE65*-related RP and LCA/EORD: 0.6% Minimum adjusted prevalence of IRD in the south-east of Norway: 1:3856
Pontikos et al. 2020 [34]	Patients with IRD Individuals: N = 4236	*RPE65*: *n* = 51 (1.2%) LCA/EORD: 3%
Sharon et al. 2020 [35]	Israeli patients with IRD Individuals: N = 3413	RP: 43% LCA/EORD: 4% *RPE65*: 1% of IRDs
Whelan et al. 2020 [36]	Irish patients with IRD N = 1004	RP: 37.75% *RPE65*: 7.41% of autosomal dominant RP LCA/EORD: 2.59% *RPE65*: 6.25% of LCA/EORD
Colombo et al. (2021) [37]	Italian patients with RP N = 591	AR-RP: 103/591 (17.5%) *RPE65*: 2/103 (2%) of AR-RP
Perea-Romero et al. (2021) [38]	Spanish patients with IRD N = 6089 individuals (4403 families)	Non-syndromic RP: 55.6% of families *RPE65*: 23/666 (3%) of families with AR-RP

AR-RP, autosomal recessive retinitis pigmentosa; EORD, early-onset retinal dystrophy; IRD, inherited retinal disease; LCA, Leber congenital amaurosis; RP, retinitis pigmentosa. * Studies were performed on diverse populations with a variety of initial diagnoses and experimental questions.

## Data Availability

Not applicable.
